# Mental Health, Risk Factors, and Social Media Use During the COVID-19 Epidemic and Cordon Sanitaire Among the Community and Health Professionals in Wuhan, China: Cross-Sectional Survey

**DOI:** 10.2196/19009

**Published:** 2020-05-12

**Authors:** Michael Y Ni, Lin Yang, Candi M C Leung, Na Li, Xiaoxin I Yao, Yishan Wang, Gabriel M Leung, Benjamin J Cowling, Qiuyan Liao

**Affiliations:** 1 School of Public Health LKS Faculty of Medicine The University of Hong Kong Hong Kong China (Hong Kong); 2 The State Key Laboratory of Brain and Cognitive Sciences The University of Hong Kong Hong Kong China (Hong Kong); 3 Healthy High Density Cities Lab HKUrbanLab The University of Hong Kong Hong Kong China (Hong Kong); 4 School of Nursing The Hong Kong Polytechnic University Hong Kong China (Hong Kong); 5 Department of Anaesthesiology Maternal and Child Health Hospital of Hubei Province Wuhan China; 6 World Health Organization Collaborating Centre for Infectious Disease Epidemiology and Control School of Public Health The University of Hong Kong Hong Kong China (Hong Kong)

**Keywords:** COVID-19, nonpharmaceutical interventions, population mental health, depression, anxiety, community, health professionals, social media, WeChat, pandemic, outbreak, public health, mental health, intervention

## Abstract

**Background:**

The mental health consequences of the coronavirus disease (COVID-19) pandemic, community-wide interventions, and social media use during a pandemic are unclear. The first and most draconian interventions have been implemented in Wuhan, China, and these countermeasures have been increasingly deployed by countries around the world.

**Objective:**

The aim of this study was to examine risk factors, including the use of social media, for probable anxiety and depression in the community and among health professionals in the epicenter, Wuhan, China.

**Methods:**

We conducted an online survey via WeChat, the most widely used social media platform in China, which was administered to 1577 community-based adults and 214 health professionals in Wuhan. Probable anxiety and probable depression were assessed by the validated Generalized Anxiety Disorder-2 (cutoff ≥3) and Patient Health Questionnaire-2 (cutoff ≥3), respectively. A multivariable logistic regression analysis was used to examine factors associated with probable anxiety and probable depression.

**Results:**

Of the 1577 community-based adults, about one-fifth of respondents reported probable anxiety (n=376, 23.84%, 95% CI 21.8-26.0) and probable depression (n=303, 19.21%, 95% CI 17.3-21.2). Similarly, of the 214 health professionals, about one-fifth of surveyed health professionals reported probable anxiety (n=47, 22.0%, 95% CI 16.6-28.1) or probable depression (n=41, 19.2%, 95% CI 14.1-25.1). Around one-third of community-based adults and health professionals spent ≥2 hours daily on COVID-19 news via social media. Close contact with individuals with COVID-19 and spending ≥2 hours daily on COVID-19 news via social media were associated with probable anxiety and depression in community-based adults. Social support was associated with less probable anxiety and depression in both health professionals and community-based adults.

**Conclusions:**

The internet could be harnessed for telemedicine and restoring daily routines, yet caution is warranted toward spending excessive time searching for COVID-19 news on social media given the infodemic and emotional contagion through online social networks. Online platforms may be used to monitor the toll of the pandemic on mental health.

## Introduction

The World Health Organization (WHO) has raised its global risk assessment of the coronavirus disease (COVID-19) to the highest level. The WHO-China Joint Mission on Coronavirus Disease 2019 has called for a worldwide response to draw on China’s extensive experience of nonpharmaceutical interventions (NPIs). NPIs aim to modify behavior to reduce the spread of infectious diseases. The first and most draconian NPIs to date have been implemented in Wuhan, China [1]. Countries around the world including France, Germany, Iran, Italy, Philippines, Kenya, Spain, the United Kingdom, and the United States have since deployed stringent interventions used in Wuhan.

**Figure 1:**
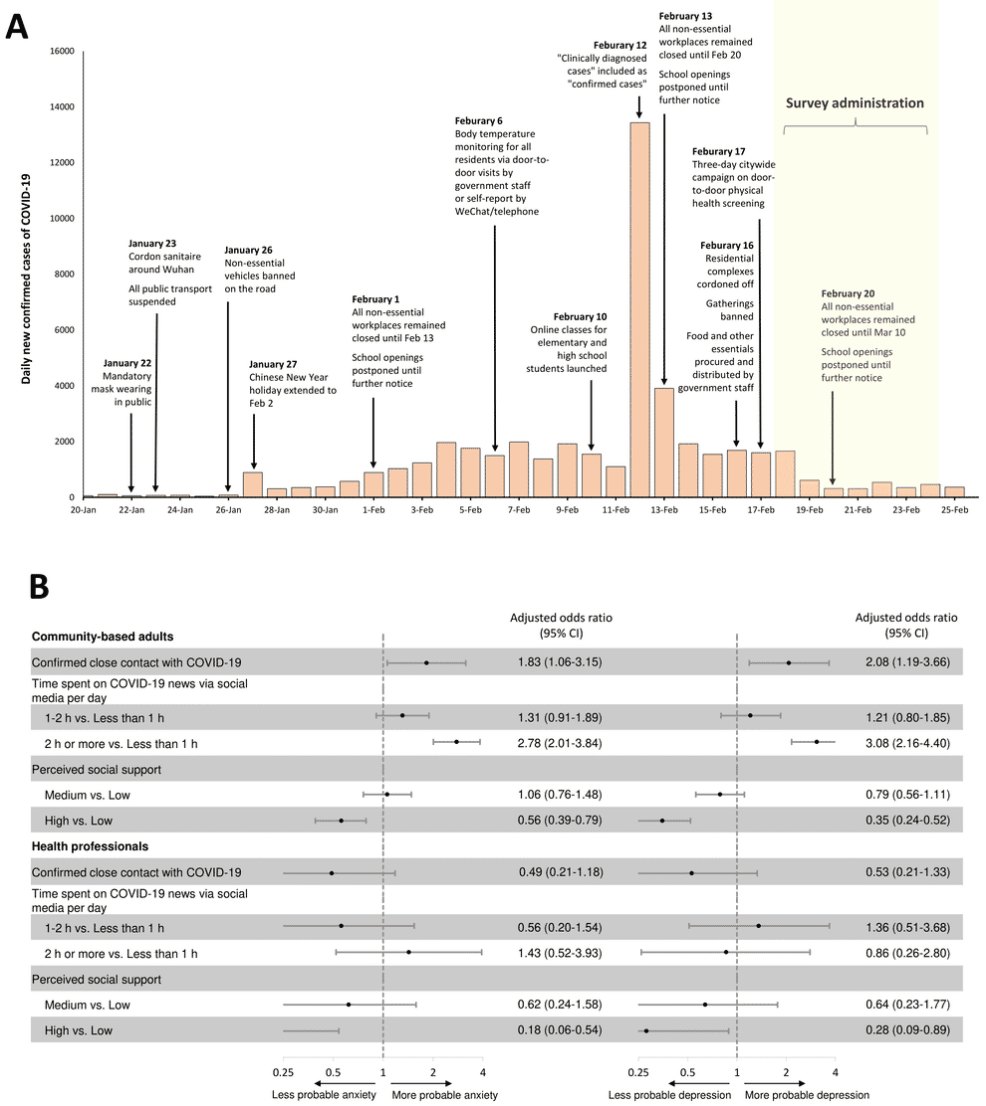
(A) Chronology of the cordon sanitaire and nonpharmaceutical interventions in Wuhan, China in 2020. The number of new confirmed cases of COVID-19 from the Health Commission of Hubei Province. (B) Risk factors of probable anxiety and probable depression during the COVID-19 epidemic and cordon sanitaire around Wuhan, China. Odds ratios are obtained through multivariable logistic regression. COVID-19: coronavirus disease.

The widespread lockdowns and stringent measures that require or encourage people to stay at home could result in more time spent on social media, particularly on searching for news or information about the pandemic [2]. Although the psychological impact of quarantine has been documented [3], the mental health consequences of the COVID-19 pandemic, community-wide NPIs, and social media use during a pandemic are unclear. Social media could mitigate the mental health impact of COVID-19 and lockdowns by maintaining social support during physical distancing, as well as providing health information, telemedicine, and online psychological counseling [4,5]. Yet social media can also spread negative emotions, rumors, and fake news during an epidemic [6-8]. In this paper, we examined risk factors including the use of social media for probable anxiety and depression in the original epicenter, Wuhan, China, and during the first month of the cordon sanitaire (Figure 1).

## Methods

### Study Design and Participants

We conducted an online survey via WeChat, the most widely used social media platform with a prevalence reaching over 90% in major cities in China [9]. We selected WeChat as the lockdown and enrollment of health professionals precluded in-person surveys and random digit dialing, respectively. Adults 18 years or older in Wuhan City during the cordon sanitaire were recruited for this study. Participants who were diagnosed with COVID-19 infections were excluded. Two sociodemographic groups, community-based adults and health professionals in Wuhan, were purposively sampled. We conducted the survey shortly after the neighborhood-level lockdown on February 16, 2020, and during the first month of the cordon sanitaire (Figure 1).

During the lockdown, residential complexes in Wuhan had established WeChat groups where at least one member from each household joins the online group to facilitate communication, support, and organize bulk purchases for daily necessities. We recruited community-based adults from 16 WeChat groups of residential complexes located in the Jianghan, Wuchang, and Jiangxia districts of Wuhan. The recruited health professionals were from 8 WeChat groups covering four main hospitals of Wuhan. We used the Tencent questionnaire system, which can collect data via WeChat and includes a built-in function to provide incentives to participants. Participants received CNY 10 (~US $1.5) via their WeChat accounts upon completion of the survey.

### Outcomes and Covariables

Probable anxiety was assessed using the validated Generalized Anxiety Disorder (GAD)-2 [10]. Total score of the GAD-2 ranges from 0 to 6 with a cut-off score ≥3, indicating probable anxiety [10].

Probable depression was assessed using the validated Patient Health Questionnaire (PHQ)-2 [11]. Total score of the PHQ-2 ranges from 0 to 6 with a cut-off score ≥3, indicating probable depression [11]. We use the term probable anxiety or probable depression as GAD-2 and PHQ-2 are brief, validated screening instruments but not diagnostic interviews.

Perceived social support was assessed using the Medical Outcomes Study Social Support Survey (MOS-SSS) [12]. The total score of the six items of the MOS-SSS ranges from 6 to 30, with a score ≤15 indicating low social support [13]. We further categorized medium and high support using a score of 16-23 and ≥24, respectively.

Demographic characteristics, exposure to COVID-19 (eg, whether they were close contacts of known cases of COVID-19 or if there were known cases of COVID-19 infections in their residential complexes), and time spent on COVID-19 news via social media and television were assessed in the survey. The study was approved by the Institutional Review Board of the University of Hong Kong/Hospital Authority Hong Kong West Cluster.

### Statistical Analysis

We estimated the prevalence of probable anxiety and probable depression in the community and health professionals with the proportion and 95% CI reported. We used multivariable logistic regression analysis to examine factors associated with probable anxiety and probable depression for these two demographic groups, giving results as adjusted odds ratios. All analyses were conducted using Stata 15.1 (StataCorp LLC).

## Results

A total of 1577 community-based adults and 214 health professionals in Wuhan completed the online survey. Among the total 1791 participants, 1341 (74.87%) were members of the selected WeChat groups. The remaining participants also reported that they were currently in Wuhan and enrolled via links of the survey forwarded by WeChat group members. Participants’ demographics are shown in Multimedia Appendix 1. As expected, a higher proportion of health professionals (n=55, 25.7%) reported being close contacts of patients with COVID-19 compared to community-based adults (n=70, 4.44%). The majority of community-based adults (n=1110, 70.39%) and health professionals (n=131, 61.2%) reported that they live in neighborhoods with COVID-19 cases. Among community-based adults, 37.16% (n=586) and 18.64% (n=294) of them spent 2 hours or more per day on COVID-19 news via social media and television, respectively. Similarly, 33.2% (n=71) and 16.4% (n=35) of health professionals spent 2 hours or more per day on COVID-19 news via social media and television, respectively.

Of the 1577 community-based adults, around one-fifth of respondents reported probable anxiety (n=376, 23.84%, 95% CI 21.8-26.0) and probable depression (n=303, 19.21%, 95% CI 17.3-21.2). Similarly, of the 214 health professionals, about one-fifth of surveyed health professionals reported probable anxiety (n=47, 22.0%, 95% CI 16.6-28.1) or probable depression (n=41, 19.2%, 95% CI 14.1-25.1). The multivariable logistic regression analysis showed that close contact with individuals with COVID-19 and spending ≥2 hours daily on COVID-19 news via social media were associated with probable anxiety and depression in community-based adults (Figure 1). In contrast, social support was associated with less probable anxiety and depression in both health professionals and community-based adults (Figure 1). Time spent on COVID-19 news via television was not associated with either probable anxiety or probable depression (Multimedia Appendices 2 and 3).

## Discussion

Our study shows that online platforms such as WeChat can be leveraged to survey community-based adults and health professionals during an epidemic and lockdown. Although our samples are not representative, in-person surveys would not be appropriate or possible during the increasingly widespread lockdowns and the COVID-19 pandemic. Random digit dialing could provide random samples in affected regions but are often associated with low response rates and would not be suitable for the enrollment of health professionals. Future studies could address these limitations by nesting follow-ups in existing random samples where available, as this could provide timely access to longitudinal population-representative data [14].

Our study showed that social support was associated with less probable anxiety and depression in both the community and health professionals. Although physical distancing is recommended to reduce the spread of COVID-19, social support should be maintained, as it is a key source of emotional support. Mitigating the mental health impact of both the pandemic and physical distancing measures is, therefore, important [15]. The internet could be harnessed for online consultation or counseling (eg, Ali Health, Ping An Good Doctor, WeChat implemented in China) and restoring daily routines (eg, online learning, telework, exercise). Yet caution is warranted toward spending excessive time searching for COVID-19 news on social media given the infodemic and emotional contagion through online social networks [8,14]. Our findings are susceptible to reverse causality, whereby anxiety or depression could lead to rumination on social media. However, the null association between time spent on television viewing and mental health suggests social media might have a specific role in adverse mental health during epidemics. Further, our findings are consistent with longitudinal studies on social media use and mental health during major population events [14,16,17].

Stress and psychiatric sequelae among health professionals requires urgent redress. This includes ensuring adequate personal protective equipment, allaying anxiety, and strengthening psychosocial support. Clinicians in turn need to be vigilant of psychiatric sequelae among patients given the potential for anxiety and depression during an epidemic [18]. COVID-19 will exact a substantial toll on the population’s well-being beyond the acute respiratory illnesses and pneumonia hospitalizations associated with infections. To mitigate the long-term impact on the populations’ physical, mental, and social well-being, monitoring the broad consequences of infections and interventions (eg, psychological, social, and economic costs), which may increasingly need to be conducted online due to widespread lockdowns and physical distancing measures, is of paramount importance. Building community resilience and, where appropriate, harnessing the social media while reducing the adverse impact should also be incorporated into global preparedness and response to pandemics.

## Appendix

Multimedia Appendix 1 Participants' demographic characteristics.

Multimedia Appendix 2 Risk factors of probable anxiety and probable depression among community-based adults during the coronavirus disease epidemic in Wuhan, China.

Multimedia Appendix 3 Risk factors of probable anxiety and probable depression among health professionals during the coronavirus disease epidemic in Wuhan, China.

## Abbreviations

COVID-19: coronavirus disease
GAD: Generalized Anxiety Disorder
MOS-SSS: Medical Outcomes Study Social Support Survey
NPI: nonpharmaceutical interventions
PHQ: Patient Health Questionnaire
WHO: World Health Organization
