# Supplementary material for: Mental Health, Risk Factors, and Social Media Use During the COVID-19 Epidemic and Cordon Sanitaire Among the Community and Health Professionals in Wuhan, China: Cross-Sectional Survey
Source: JMIR Ment Health. 2020 May 12;7(5):e19009. doi: 10.2196/19009 (PMC7219721; doi:10.2196/19009)
Supplement: Multimedia Appendix 1 [file mental_v7i5e19009_app1.pdf]

	Community-based adults	Health professionals
**Sex**		
Men	39.2%	31.2%
Women	60.8%	68.8%
**Age group (Years)**		
18-34	38.6%	58.9%
35-44	28.4%	33.6%
45 or above	33.0%	7.5%
**Marital status**		
Married	68.2%	66.4%
Never married	24.7%	31.3%
Widowed, divorced or separated	7.1%	2.3%
**Educational attainment**		
Secondary or below	29.1%	3.7%
Tertiary	71.8%	96.3%
**Employment**		
Economically inactive or unemployed	38.7%	0.0%
Employed	61.3%	100.0%
**Health profession**		
Nurse	N/A	50.5%
Doctor	N/A	37.8%
Others (e.g. Pharmacist, nurse assistant)	N/A	11.6%
**Monthly income (CNY)**		
<4,000	34.4%	12.7%
4,000-5,999	27.4%	19.0%
6,000 or more	38.1%	68.2%
**Confirmed close contact with COVID-19**		
No	95.6%	74.3%
Yes	4.4%	25.7%
**Living in a neighbourhood with COVID-19 cases**		
No	29.6%	38.8%
Yes	70.4%	61.2%
