# Supplementary material for: Mental Health, Risk Factors, and Social Media Use During the COVID-19 Epidemic and Cordon Sanitaire Among the Community and Health Professionals in Wuhan, China: Cross-Sectional Survey
Source: JMIR Ment Health. 2020 May 12;7(5):e19009. doi: 10.2196/19009 (PMC7219721; doi:10.2196/19009)
Supplement: Multimedia Appendix 2 [file mental_v7i5e19009_app2.pdf]

	Probable anxiety	Probable depression
**Sex**		
Men	1 (ref)	1 (ref)
Women	1.11 (0.85-1.45)	1.14 (0.85-1.52)
**Age group (Years)**		
18-34	1 (ref)	1 (ref)
35-44	1.18 (0.83-1.68)	1.23 (0.84-1.78)
45 or above	0.96 (0.65-1.40)	**0.62 (0.40-0.95)**
**Marital status**		
Married	1 (ref)	1 (ref)
Never married	0.96 (0.66-1.39)	1.21 (0.82-1.79)
Widowed, divorced or separated	1.15 (0.73-1.80)	1.00 (0.60-1.68)
**Educational attainment**		
Secondary or below	1 (ref)	1 (ref)
Tertiary	1.07 (0.78-1.47)	1.05 (0.73-1.49)
**Employment**		
Economically inactive or unemployed	1 (ref)	1 (ref)
Employed	0.88 (0.64-1.19)	0.79 (0.56-1.12)
**Monthly income (CNY)**		
<4,000	1 (ref)	1 (ref)
4,000-5,999	0.87 (0.62-1.21)	0.72 (0.49-1.05)
6,000 or more	**0.71 (0.51-0.99)**	0.80 (0.56-1.15)
**Confirmed close contact with COVID-19**		
No	1 (ref)	1 (ref)
Yes	**1.83 (1.06-3.15)**	**2.08 (1.19-3.66)**
**Living in a neighbourhood with COVID-19 cases**		
No	1 (ref)	1 (ref)
Yes	1.29 (0.97-1.73)	**1.53 (1.11-2.12)**
**Time spent on COVID-19 news on social media per day**		
Less than 1 hour	1 (ref)	1 (ref)
1-2 hours	1.31 (0.91-1.89)	1.21 (0.80-1.85)
2 hours or more	**2.78 (2.01-3.84)**	**3.08 (2.16-4.40)**
**Time spent on COVID-19 news on TV per day**		
Less than 1 hour	1 (ref)	1 (ref)
1-2 hours	1.09 (0.77-1.54)	0.88 (0.60-1.31)
2 hours or more	1.08 (0.76-1.53)	1.15 (0.79-1.67)
**Social support**		
Low (15 or below)	1 (ref)	1 (ref)
Medium (16-23)	1.06 (0.76-1.48)	0.79 (0.56-1.11)
High (24-30)	**0.56 (0.39-0.79)**	**0.35 (0.24-0.52)**

Note: Social support was measured by the Medical Outcomes Study Social Support Survey.
