# Supplementary material for: Mental Health, Risk Factors, and Social Media Use During the COVID-19 Epidemic and Cordon Sanitaire Among the Community and Health Professionals in Wuhan, China: Cross-Sectional Survey
Source: JMIR Ment Health. 2020 May 12;7(5):e19009. doi: 10.2196/19009 (PMC7219721; doi:10.2196/19009)
Supplement: Multimedia Appendix 3 [file mental_v7i5e19009_app3.pdf]

	Probable anxiety	Probable depression
**Sex**		
Men	1 (ref)	1 (ref)
Women	0.81 (0.38-1.75)	0.60 (0.26-1.36)
**Age group (Years)**		
18-34	1 (ref)	1 (ref)
35-44	1.32 (0.57-3.01)	1.05 (0.44-2.47)
45 or above	1.68 (0.46-6.17)	0.22 (0.03-1.90)
**Monthly income (CNY)**		
<4,000	1 (ref)	1 (ref)
4,000-5,999	0.75 (0.22-2.54)	1.15 (0.33-4.02)
6,000 or more	0.63 (0.22-1.84)	0.90 (0.29-2.73)
**Confirmed close contact with COVID-19**		
No	1 (ref)	1 (ref)
Yes	0.49 (0.21-1.18)	0.53 (0.21-1.33)
**Living in a neighbourhood with COVID-19 cases**		
No	1 (ref)	1 (ref)
Yes	1.08 (0.49-2.36)	1.17 (0.51-2.68)
**Time spent on COVID-19 news on social media per day**		
Less than 1 hour	1 (ref)	1 (ref)
1-2 hours	0.56 (0.20-1.54)	1.36 (0.51-3.68)
2 hours or more	1.43 (0.52-3.93)	0.86 (0.26-2.80)
**Time spent on COVID-19 news on TV per day**		
Less than 1 hour	1 (ref)	1 (ref)
1-2 hours	0.97 (0.38-2.45)	1.44 (0.55-3.77)
2 hours or more	0.76 (0.24-2.37)	2.70 (0.81-8.98)
**Social support**		
Low (15 or below)	1 (ref)	1 (ref)
Medium (16-23)	0.62 (0.24-1.58)	0.64 (0.23-1.77)
High (24-30)	**0.18 (0.06-0.54)**	**0.28 (0.09-0.89)**

Note: Covariables were not included in the multivariable logistic regression models due to demographic homogeneity or insufficient samples. Social support was measured by the Medical Outcomes Study Social Support Survey.
